# Determinants of Sexual Network Structure and Their Impact on Cumulative Network Measures

**DOI:** 10.1371/journal.pcbi.1002470

**Published:** 2012-04-26

**Authors:** Boris V. Schmid, Mirjam Kretzschmar

**Affiliations:** 1 Unit Epidemiology & Surveillance, Centre for Infectious Disease Control, National Institute of Public Health and the Environment (RIVM), Bilthoven, The Netherlands; 2 Julius Centre for Health Sciences & Primary Care, University Medical Centre Utrecht, Utrecht, The Netherlands; Pennsylvania State University, United States of America

## Abstract

There are four major quantities that are measured in sexual behavior surveys that are thought to be especially relevant for the performance of sexual network models in terms of disease transmission. These are (i) the cumulative distribution of lifetime number of partners, (ii) the distribution of partnership durations, (iii) the distribution of gap lengths between partnerships, and (iv) the number of recent partners. Fitting a network model to these quantities as measured in sexual behavior surveys is expected to result in a good description of *Chlamydia trachomatis* transmission in terms of the heterogeneity of the distribution of infection in the population. Here we present a simulation model of a sexual contact network, in which we explored the role of behavioral heterogeneity of simulated individuals on the ability of the model to reproduce population-level sexual survey data from the Netherlands and UK. We find that a high level of heterogeneity in the ability of individuals to acquire and maintain (additional) partners strongly facilitates the ability of the model to accurately simulate the powerlaw-like distribution of the lifetime number of partners, and the age at which these partnerships were accumulated, as surveyed in actual sexual contact networks. Other sexual network features, such as the gap length between partnerships and the partnership duration, could–at the current level of detail of sexual survey data against which they were compared–be accurately modeled by a constant value (for transitional concurrency) and by exponential distributions (for partnership duration). Furthermore, we observe that epidemiological measures on disease prevalence in survey data can be used as a powerful tool for building accurate sexual contact networks, as these measures provide information on the level of mixing between individuals of different levels of sexual activity in the population, a parameter that is hard to acquire through surveying individuals.

## Author Summary

Although many diseases spread so easily between humans that someone could be infected by any of his or her daily social contacts, such is not the case for sexually transmitted diseases. Most of us have a very limited number of concurrently ongoing sexual partnerships, and thus the contact network over which sexually transmitted diseases spread tends to be very sparsely connected. The exact structure of these sexual networks plays an important role in how easy and fast sexually transmitted diseases spread through a population, and how effective various health care interventions will be. In this paper we use a simulation model to understand how the collective sexual behaviour of individuals relates to the summary measures of network structure (such as “lifetime number of partners”, and “duration of previous partnership”) that are typically used to build models of disease transmission over sexual networks. Based on our understanding of this relationship, we simulated sexual networks which have summary measures of network structure that are very similar to that of a real sexual network. Using these networks in disease transmission models will increase our ability to predict the effectiveness of health care interventions.

## Introduction

The transmission dynamics and epidemiology of sexually transmitted infections (STI) are shaped by the sexual network through which they propagate. Sexual networks are characterized by their dynamic nature and large heterogeneity that reflects the diversity of human sexual behavior. For long now, mathematical modelers have attempted to describe the essential features of that behavior and the resulting networks in various types of models in order to understand the infection dynamics and possible impact of STI interventions [1]–[6]. It has remained extremely challenging to take all aspects that determine the structure of a sexual network into account in a comprehensive way, while at the same time not cluttering models with too much detail that makes them difficult to handle. One approach has been the design of individual based simulation models that follow certain algorithmic rules to describe the formation and dissolution of partnerships explicitly [7]–[11]. This approach has the advantage above models that assume a mass-action style of mixing between individuals, that partnerships of different durations are explicitly present in the model, and thus that important aspects of *Chlamydia trachomatis (Ct)* transmission dynamics, such as the duration of the gap/overlap between sequential partnerships [12]–[14], and re-infection events between partners [15], [16] are not ignored. Individual based models have proven to be a flexible and useful tool in this regard.

In designing an individual based model, decisions have to be taken in how to implement partnership formation and dissolution and intervention strategies in terms of simple rules that can be coded into a computer program. While striving at parsimony and simplicity in order to be able to understand the resulting dynamics, one also wants to capture the essential features of human sexual behavior that impact on the transmission dynamics and intervention effectiveness of STI. By doing that one usually validates the model using aggregate data for some quantities describing sexual behavior such as lifetime number of partners, or partnership duration. One can analyze how well models are able to reflect those summary measures of sexual behavior and consequently the distribution of STI prevalence in a population [11]. However, it is also known that macrostructure of an individual based model does not uniquely determine the microstructure of a sexual network [17] and consequently models with similar macrostructures can lead to different results about intervention impact [18].

There are four major quantities that are routinely measured in sexual behavior surveys that are thought to be especially relevant for the performance of sexual networks in terms of disease transmission. These are (i) the cumulative distribution of lifetime number of partners [19], [20], (ii) the distribution of partnership durations [14], [20], (iii) the distribution of gap lengths between partnerships [14], [20], [21], and (iv) the distribution of the number of recent partners [11], [22]. Fitting a network model to these quantities as measured in sexual behavior surveys is expected to result in a good description of *Ct* transmission in terms of the heterogeneity of the distribution of the infection within a population [11].

In this paper we investigate how these population level summary measures of sexual activity relate to the underlying sexual behaviors of the population and their heterogeneity on the individual level. We use an individual based simulation model, in which pair formation and separation are described as a dynamic process. The model is based on an earlier model of Kretzschmar et. al. [7], but has been extensively restructured to accommodate for more detail and heterogeneity in individual behaviour. Central to the model implementation is the function that describes the number of partnerships that an individual can simultaneously maintain during different periods of his/her life. This changing “capacity” of individuals controls their onset of sexual availability, as well as their propensity for acquiring concurrent partnerships. In addition, we demonstrate how including heterogeneity of sexual behavior on the individual level improved the performance of the model in describing population level measures of sexual behavior.

## Results

The sexual network model presented here consists of a heterosexual population of 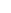
50,000 individuals, uniformly distributed over the ages 13 to 64. Connections in the network represent sexual partnerships between individuals. The network is dynamic: partnerships are continuously formed and dissolved. The model keeps the population size constant over the 40 years of simulation by adding young individuals to the network at age 13, as old individuals retire from the network (by no longer forming new partnerships) when reaching age 65. Individuals in the model are defined by their date of birth, gender, their current partnership(s), the maximum number of partners that they can concurrently maintain (i.e., their “capacity”), and their *Chlamydia trachomatis* infection status. In this model we studied how individual heterogeneity influences population-level summary measures of the sexual contact network.

### Cumulative lifetime number of partners

**Figure 1:**
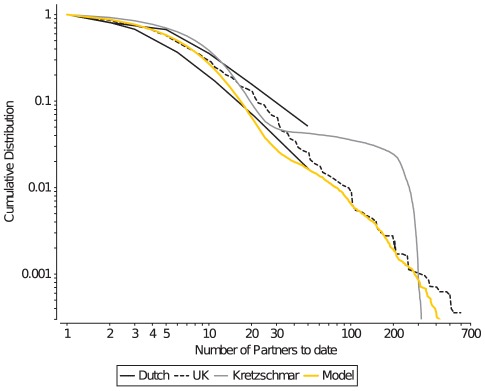
Cumulative plots of the lifetime number of partnerships (CLNP). Empirical data on the CLNP is available as a range for the Dutch sexual survey data (black solid lines), and as absolute numbers for the UK survey data (black dashed line). The Kretzschmar model (gray lines) struggles in reproducing the powerlaw-like distribution of the survey data, whereas the current model can closely reproduce such a distribution (orange lines).

The large heterogeneity of individuals in their sexual behaviour is perhaps most apparent in the distribution of the number of partners that individuals in a population have had partnerships with. This lifetime number of partnerships ranges from 1 to 600–1000 partners for sexually active individuals [11], [19], [20], and is commonly presented as a cumulative lifetime number of partnerships (CLNP) plot. One of the remarkable features of the CLNP is that the higher end of its distribution (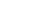 partners) has a powerlaw-like distribution [19] (Fig. 1).

Whether simulation models can accurately reproduce the heterogeneity in the lifetime number of partners depends on their implementation of the “core group”: a label introduced by Yorke et. al. in 1978 [23], [24] for sexually highly active individuals that have many, possibly concurrent partnerships in a short period of time. Many models struggle with reproducing the powerlaw-like distribution of the CLNP, and typically end up with a distribution that reflects the two or three levels of sexual activity defined in these models (e.g., moderate, intermediate, and core-group individuals [7], [9]–[11]).

We concluded that behavioural heterogeneity in sexually highly active individuals is underestimated in models that distinguish a limited number of sexual activity levels, even if there is stochastic variation among individuals. Rather than using a limited number of sexual activity levels, we correlated the capacity for concurrent partnerships in core-group individuals with two pre-determined characteristics of high risk behaviour, namely the onset and the duration of the core-group period for an individual (Table 1). The earlier the onset, and the longer the duration, the higher would be their maximum number of concurrent partnerships (see methods for details). Furthermore, we controlled the total number of partnerships within the moderate group and within the core-group separately (Table 2). This allowed us to independently regulate how close the number of partnerships within each group would be to the maximum number of partnerships that that group could maintain (i.e. the sum of their capacities). For example, a high number of partnerships in the core-group, relative to the sum of their capacities would result in high lifetime number of partnerships per core-group member without affecting the rate at which moderate individuals acquired new partners. These adjustments gave us considerable control on the shape of the CLNP plot (Fig. 1), and resulted in a powerlaw-like distribution of the CLNP that is similar to that of real sexual contact networks [11], [19], [20].

**Table 1:** Model parameters for constructing capacity vectors.

Parameter	Subcategory	Value
Onset of sexual availability (OSA)	all	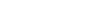
Onset of core-group behaviour (OC)	men	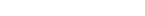
	women	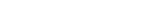
Duration of core-group behaviour (DC)	men	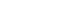
	women	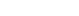
Capacity of moderate individuals	all	0 prior to OSA, 1 after OSA
Capacity level function for core-group	men	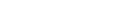
	women	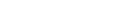
Capacity after core-group period	max capacity 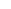 4	2
	otherwise	1

**Table 2:** Model parameters at the population level.

Parameter	Category	Value
Population size		50,000 individuals between age 13–64
Number of partnerships	moderate men - moderate women	17,750 (35.5%)
	core men - core women	150 (0.3%)
	core men - moderate women	1650 (3.3%)
	moderate men - core women	500 (1%)
Fraction core during lifetime	men	7%
	women	5%
Mean duration 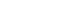 a	short-term	13 1/3 days
	medium-term	150 days
	long-term	5882 days (16.1 years)
Model 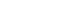 b		4 days
Age-dependent chance of participating in the pair formation process (for moderates only)	men	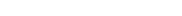
	women	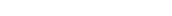

a Durations of partnerships are sampled from an exponential distribution with the listed mean, and a minimum of 1 day.

b Individuals are capable of acquiring multiple partnerships in a single timestep.

### Number of recent partners

The current model accurately replicates the CLNP observed in real sexual contact networks. However, similar CLNP distributions can be the result of very different age distributions at which the individuals acquire most of their lifetime number of partners [17]. This was recently demonstrated in a comparison study of three models of sexual contact networks [11]. Because the age at which individuals are exposed to many sexual partners is also the age at which they are most vulnerable for contracting, as well as most efficient at transmitting STIs [11], [22], it is necessary for sexual network models to accurately simulate the distribution of recent number of partners for different age groups.

**Figure 2:**
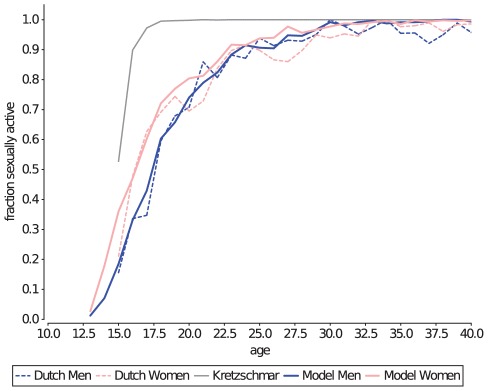
the age of sexual debut is the result of age of sexual availability, and the time it takes to find a first sexual partner. Data from the Dutch sexual survey is plotted in for both genders (dashed lines), and the model output (thick lines). The Kretzschmar model (gray line) results in a too-early age of sexual debut for both men and women (for clarity plotted together as one line).

**Figure 3:**
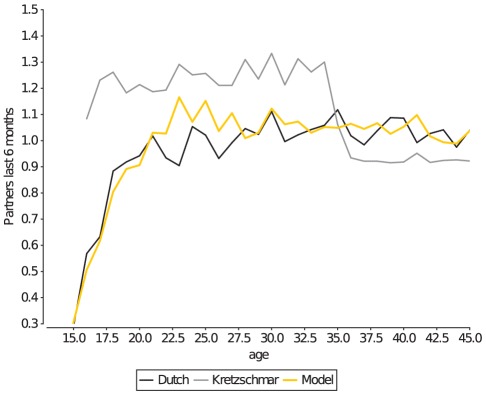
Mean number of partners in the last 6 months. This measurement is indicative of the age at which individuals accumulate most of their lifetime number of partnerships. The early age of sexual debut and early onset of core-group behaviour results in an overestimation of the mean number of partners in the Kretzschmar model (gray line), which was resolved in the current model by adding host heterogeneity in the onset of availability for sexual partnerships and onset and duration of core-group membership (orange line).

In the model described by Kretzschmar et. al. in 1996 [7], all individuals were available for sexual partnership from the moment they entered the population at age 15, and 5% of these individuals were labeled as core-group members, and will remain so until the age of 35. The homogeneous age of sexual debut and onset of core-group behaviour of individuals results in a premature age of sexual debut (Fig. 2, gray line), and an overestimation of the mean number of recent partners in the population younger than 35 (Fig. 3 gray line). By adding heterogeneity in the age at which individuals become available for sexual partnerships, and (if applicable) in the onset and duration of their core-group period (Table 1), the current model could accurately match the observed mean number of recent partners per age-group (Fig. 3 orange line).

**Figure 4:**
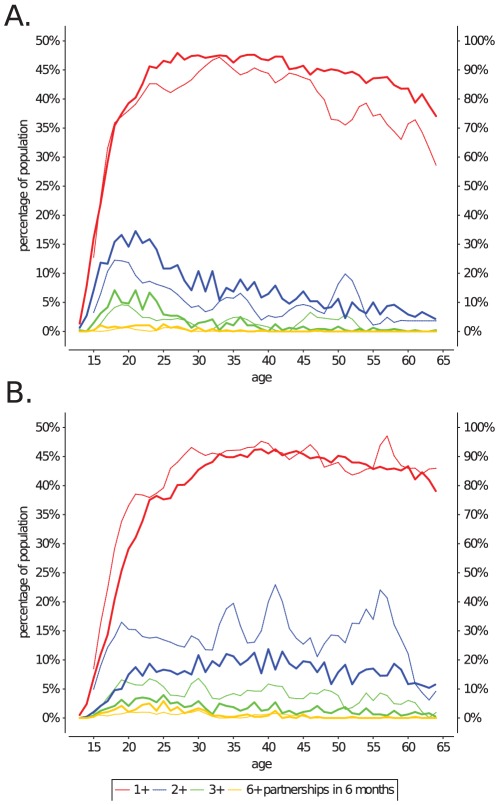
Number of partners in the last 6 months, stratified by age, gender, and sexual activity. Panel A shows the number of recent partners for men, and panel B for women. Stratification of the number of partners by sexual activity give a detailed insight in many aspects of a sexual networks' structure. The percentage of people with 1+ partners is given on the right-hand axis, and the percentage for 2+, 3+ and 6+ partners is given on the left-hand axis.

As the level of sexual activity is such an important indicator of STD risk, we further stratified the recent sexual activity of the Dutch population into the fraction of the population with 1+, 2+, 3+ or 6+ partners in the last half year, as well as by age and gender (Fig. 4). We found that the current model could match many of the features of this more detailed measure of recent sexual activity of the Dutch population. Furthermore, at this level of detail, the recent number of sexual partners appears to be sensitive to most of the model parameters. It can therefore serve as an excellent measure to validate a sexual network model on, but at the same time is difficult to interpret how it depends on these model parameters. What follows is a discussion of how different parts of Fig. 4 relate to the underlying model mechanics.

The fit of the model for individuals with 1+ partnership is predominantly determined by the difference between the total number of partnerships that the model maintains in the moderate group of individuals (Table 2), and the maximum number of partnerships that this group can maintain, given their capacity. If the difference between the two is small individuals will quickly find partners, and few individuals will have been without partner in the last 6 months. A second important factor that influences the fit for individuals with 1+ as well as for those with 2+ partnerships is the duration and relative frequency of different partnership types. The model defines three types of partnerships (short-, medium-, and long-term), all of which have exponentially distributed durations, but with different average lengths of a partnership (see next section, and methods). If, for example, the relative frequency of short-term partnerships is increased, more of the moderate individuals can acquire 1, 2 or even 3+ partnerships in 6 months. Finally, transitional concurrency [25] is a third factor that influences the recent number of partners of moderate individuals. Transitional concurrency is the overlap between the end of one partnership and the beginning of the next. Transitional concurrency is implemented in the model by defining the cost of a partnership as 0 as it nears the end of its duration, thus freeing up “capacity” for the individual (see section on gap length, and methods). If transitional concurrency is set to become possible earlier during partnerships, it will increase the number of moderate individuals that have had 2+ recent partner, but also increases the number of moderate individuals that had no partnerships in the last 6 months.

The group of 3+ recent partnerships (Fig. 4 green lines) past age 30 is predominantly formed by a fraction of the former core-group members that continues to keep an increased capacity (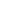) after their main period of core-group behaviour (Table 1). Many moderate individuals will by chance be locked into a long-term partnership at that age, and thus have few opportunities to accumulate recent partners. The group with 6+ recent partnerships is almost exclusively defined by the characteristics of the core-group, i.e. its overall size, the age at which core-group behaviour starts, its duration, and how many partnerships the model maintains within the core-group, compares to the sum of the capacities of that group (Table 1,2).

### Partnership duration

**Figure 5:**
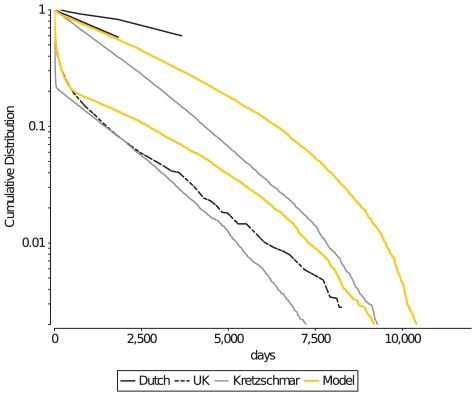
Duration of current and second-most recent partnership. The distribution of partnership duration of the current partnership (solid black lines, Dutch data), and of the second-most recent partnership (dashed black line, UK data). The Kretzschmar model has only short- and long-term partnerships, and thus provides a less precise fit to the data. The discrepancy between the UK data on long-term partnership duration and the model (bottom orange line) is the consequence of fitting the model to both the UK second-most recent partnership data and the Dutch survey data on the duration of the current partnership (top orange line).

Partnership duration has been recorded in limited detail and only for the current partner in the Dutch sexual survey (Fig. 5), and thus predominantly reflects the duration of long-term partnerships in the Netherlands. Therefore, we also use the more detailed Natsal 2000 UK survey data [11], [26] to fit the model, which also includes information on the duration of previous partnerships. From the UK survey data it appears that there are three typical durations of partnerships (short-, medium- and long-term, Fig. 5 dashed black line), each of which is exponentially distributed (Table 2).

As mentioned in the previous section, partnership duration (or put more precisely, the relative frequency of long-term partnerships) predominantly affects the number of recent partners of the moderate population; a high relative frequency of long-term partnerships means that many moderate individuals will have had only 1 sexual contact in the last 6 months. Partnership duration has less effect on the number of recent partners of core-group individuals, because core-group members have a capacity of 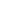, and are limited to a maximum of 2 concurrent long-term partnerships (see methods). Therefore, core-group members always have capacity available for at least one short or medium-term partnership.

In the model there are no individual sexual behaviour that directly affect the duration of partnerships: durations are determined when a partnership forms, and are independent of the further actions of the individuals involved (such as concurrency). One small exception is that individuals that stop being core-group members have to reduce their current number of concurrent partnerships to match their new maximum capacity (see methods). We conclude that partnership duration shape the sexual network relatively independent from the distribution of the number of partnerships of individuals in the population.

### Gap length

The time between sequential partnerships (e.g. the gap length [21]) is an important factor related to *Ct* prevalence [12]–[14]: negative gap length (e.g. an overlap in partnerships) signals the amount of concurrency in the population: in contrast to serial monogamy, concurrent partnerships provide a two-way channel for *Ct* to spread, and not just from a previous partner to a new partner. In contrast, positive gap length indicates the chance that an individual has had to spontaneously clear *Ct* prior to engaging in a new partnership [13]. The precise definition of gap length is the “time between the end of the second most recent partnership, and the start of the most recent partnership”, in which the order of recentness is determined by the last date that partnerships were still ongoing. Where two or more partnerships are tied for recentness, their order is randomly determined.

**Figure 6:**
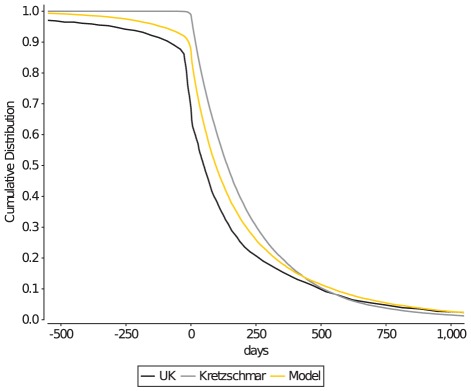
Gap length, i.e. the time between the two most recent partnerships. Negative gaps indicate overlapping partnerships, whereas positive gaps indicate the time it takes to acquire a new partner. The current model (orange line) does not fully match the UK survey data (black line). It would require a higher level of transitional concurrency in the simulated population, but one that is incompatible with the Dutch survey data on Fig. 6.

**Figure 7:**
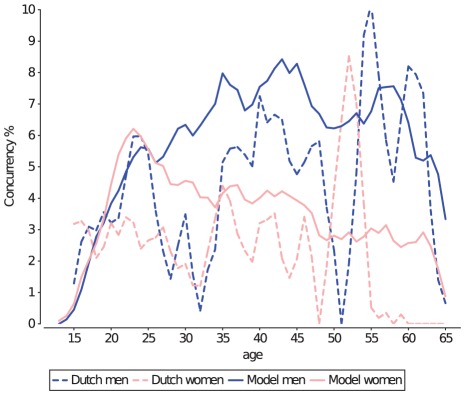
Transitional concurrency in partnerships that are less than 6 months old. The model (solid lines) matches Dutch sexual survey data (dashed lines) well when transitional concurrency is possible during the last 15% of a partnership (from a man's perspective), or the last 7.5% of a partnership from a woman's perspective. The y-axis shows the percentage of individuals with a medium- or long-term partnerships that is less than 6 months old, which had a period of transitional concurrency during this partnership.

The Dutch sexual survey data has insufficient information to reconstruct gap lengths, so the current model was fitted to UK survey data on gap length [11], [20] (Fig. 6), and subsequently adjusted as to fit Dutch survey data on transitional concurrency in recently started (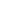 months) steady partnerships (Fig. 7), where “steady” was interpreted as being either a medium-term or a long-term partnership.

About 3% of the UK population reports a negative gap length of more than 500 days between the last two partnerships (Fig. 6, black line), and up to 35% of the population has had overlap between his/her last two partnerships. These percentages indicate that a model of a sexual contact network should allow concurrency of long-term partnerships (to see overlaps of 500+ days), and should not limit the occurrence of concurrent partnerships to the small core-group of the population, as was the case in the Kretzschmar model. The current model allows transitional concurrency [25] during the last 15% of a partnership for men, and the last 7.5% for women (Fig. 6, orange line). The positive gap length in the UK is relatively short compared to the duration of an untreated *Ct* infection: only 15% of the population takes more than 1 year to find a new partner.

In the model, the positive gap length is determined by the chance of individuals to participate in the pair-formation process for each timestep (Table 2), and by the total number of partnerships that the model tries to maintain: if either is too low, the time between partnerships (i.e. positive gap size) increases. Positive gap length is also affected by transitional concurrency, as an increase in concurrency increases the fraction of the population that is available for new partnerships, and thus increases the competition for those that are single and seeking a new partnership. The result is that the average positive gap length becomes larger with higher levels of transitional concurrency.

### *Ct* prevalence distribution

**Figure 8:**
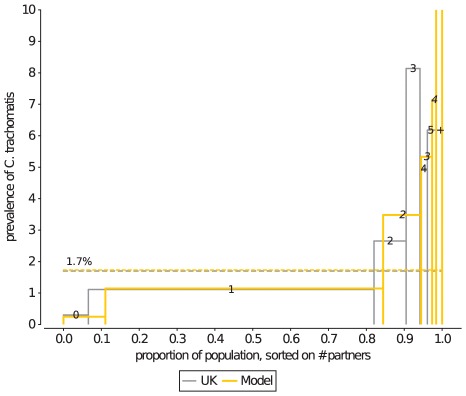
The distribution of Ct prevalence, stratified by number of partners in the last year. Although the current model is fitted to the Dutch sexual contact network, the *Ct* prevalence distribution for those with 0, 1, and 2 partners in the last year corresponds well to the UK survey data. At higher levels of sexual activity, the model underestimates the *Ct* prevalence in individuals with an intermediate level sexual activity, and overestimates the *Ct* prevalence of the core group. The Ct prevalence in the highest category for the model is 22%. The *Ct* distribution shown here is the average of 15 years of sampling from 10 instances of the sexual contact network.

The distribution of *Ct* prevalence in the population, stratified by level of sexual activity was introduced as a new summary measure by Althaus et. al. [11] that combines sexual behaviour with epidemiological data. As expected, the *Ct* prevalence within a stratum rises as the level of sexual activity of its individuals rises from 0 to 3 partners in the last year (Fig. 8). Remarkably, however, is that in the UK survey data the *Ct* prevalence in the subsequent strata (4 and 5+ partners) drops again to the prevalence levels halfway between that of the strata of 2 and 3 partners per year. Neither the current model, nor earlier sexual contact network models [11] are capable of reproducing this observation, or shed light on the mechanism behind it. Two possible mechanisms are explored in Supporting Text S1, namely 1) that prolonged and frequent exposure to *Ct* could result in a protective immune response [27]–[30], and thus reduce the *Ct* prevalence in those population groups that are most likely to have experienced a prolonged infection, and 2) that due to coital dilution, that is the decrease in coital frequency when maintaining concurrent partnerships, those with concurrent partnerships will have a reduced chance of acquiring *Ct* [31]–[33].

A second important feature of the distribution of *Ct* prevalence in the population is that it provides information about the amount of mixing between moderate and core-group individuals in the population. The level of assortative mixing is an important measure for disease spread [13], [24], but is difficult to measure in surveys, as the surveyed individuals need to have accurate knowledge about the life history of their (possibly short-term) partners. Therefore, models of sexual contact networks typically rely on assumptions on the level of mixing [7], [9], [11]. However, as *Ct* becomes more concentrated in individuals with a high level of sexual activity [2], [13], [23]

(Fig. 8), in strongly assortative populations, and less so in more well-mixed populations, one can use the difference between the average *Ct* prevalence of the population, and the *Ct* prevalence of for example the group of individuals with 1 partnership in the last year, as an observation against which to fit the amount of assortative mixing in the model.

The level of mixing on sexual activity in the model (Table 2) that well describes the UK survey data is a situation in which 87% of the partnerships of the core-group are with moderate members. If we define core-group members as those individuals with 5+ partnerships in the last 12 months, the model results are comparable to earlier investigations on mixing of sexual activity [34], [35], and the amount of mixing in the model can be characterized as having a moderate assortativity coefficient of 0.25 [35].

## Discussion

In this paper we investigated how four population level summary measures of sexual activity, are related to the underlying sexual behaviours of individuals, and to the heterogeneity in behaviour on the individual level. We showed that the “recent number of sexual partners” summary measure is very sensitive to many of the sexual characteristics of simulated individuals, whereas “gap length” and “partnership duration” are predominantly defined by homogeneous traits in the model (i.e. the amount of transitional concurrency, and the relative frequency and duration of short, medium and long-term partnerships). Heterogeneity of sexual behaviour in individuals, and especially the heterogeneity in sexual capacity of core-group individuals, was found to play a large role in the summary measure “cumulative lifetime number of partners”, and instrumental in recreating the measure's powerlaw-like distribution [19]. We therefore conclude that an extensive heterogeneity in the behaviour of individuals in terms of acquiring partners, is likely to play an important role in the network structure of real sexual networks. Whether heterogeneity in individual behaviour plays a similar role for the summary measures of gap length and partnership duration is undecided: we find that, given the level of detail with which we studied these two summary measures, we could accurately recreate them in simulation models using homogeneous descriptions of sexual behaviour. However, a more detailed study of these summary measures is necessary (for example by stratifying by age and gender, or by studying them in terms of the life history of individuals) to conclude whether heterogeneity in behaviour in transitional concurrency and in partnership duration plays an important role in the structure of sexual networks.

The summary measure introduced by Althaus et. al. [11] that describes the distribution of *Ct* prevalence in the population highlight our incomplete understanding of disease transmission through sexual networks. We currently do not know whether the lower than expected *Ct* prevalence in individuals with the highest levels of sexual activity [11] is a feature of the structure of real sexual networks, or that it is related to some form of protective immunity upon frequent exposure to, but not necessarily infection with *Ct* [30]. The summary measure has an additional important quality: the distribution of *Ct* prevalence in a population can be used to inform sexual network models on the degree of mixing between individuals of different levels of sexual activity. This parameter is known to be very important for the structure of sexual contact networks [13], [35], but is unfortunately not present in current sexual behaviour survey data (for ways to set up an unbiased sexual survey of mixing patterns, see Boily et. al [36]). In this paper we presented a clear way, based on existing theory [2], [13], [23], how to indirectly extract the mixing between individuals with different levels of sexual activity from disease prevalence distributions.

As sexual network models are now routinely used throughout the world to determine both the feasibility and cost-effectiveness of nation-wide healthcare interventions [37]–[39], it is critical that these models move towards a state where they are able to make accurate predictions. By increasing our understanding of the complex relations between population-level summary measures, and the heterogeneity of individual sexual behaviour, we were able to make large qualitative improvements in reproducing sexual network summary measures in comparison to earlier versions of sexual network models [11], [18], at the price of a moderate amount of additional complexity.

In conclusion, shifting the models' perspective from a population-level description to that of the heterogeneity in individual sexual behaviour opened up new ways to fit sexual contact network models to sexual survey data. In the future, a modeling approach in which sexual network structure more and more emerges from individuals' sexual behavior as studied in social psychology may further improve the realism of sexual network models.

## Methods

### Dutch and UK sexual survey data

The population-level summary measures are based on data from the Rutgers Nisso Group Dutch population survey on sexual health in 2009 [40]. This survey entails 6428 individuals that are weighted on gender, age, ethnicity, and the degree of urbanization of their hometown. Individuals that fell outside the age-range of 13–64 were excluded from the data, as were those that reported having had homosexual partnerships, that were paid for partnerships, or had included prostitute visits in their answers on sexual activity. This procedure left a total of 5402 individuals (Table 3). For population-level summary measures which could not be derived from the Dutch sexual survey, we used the Natsal 2000 UK sexual survey [26] as presented in Althaus et. al. [11].

**Table 3:** Dutch sexual survey summary data.

Category	Gender	Subcategory	Count	Mean, Range
Total			5402	
By gender	Men		2499	
	Women		2903	
By age	Men	15–44 y		69, 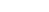 a
		45–64 y		18, 11–35
By age	Women	15–44 y		81, 55–140
		5–64 y		20, 11–40
By sexual activity ( 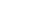 )	Men	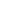 b		55, 11–17
		2+		10, 3–17
		3+		3.7, 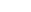 c
		6+		1.3, 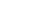 c
By sexual activity ( 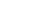 )	Women	1+		66, 18–104
		2+		5.7, 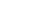 c
		3+		2.5, 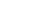 c
		6+		1, 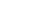 c

a Indicates mean and minimum-maximum number of individuals in this category.

b 1+ is defined as having 1 or more partners in the last six months.

c data is not available for all ages due to small sample size.

All Dutch survey population measures presented in the manuscript take into account the weighted value of individuals (e.g. individuals that had a weight 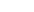 associated with them because they were sampled from an underrepresented population group also contribute 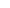 times as much to the population level summary measures as individuals with a weight of 1). The population measure on “sexual activity in the last 6 months” and the model data (Fig. 4) were smoothened to facilitate a visual comparison, using a Savitzky-Golay non-linear smoothing filter [41], [42] (parameters: window size 5, coefficient 4, left-padding 0, right-padding recent mean for 1+ and 2+ partners, and 0 for 3+ and 6+ partners). The smoothing filter works similar to a running average, but performs better at preserving the trends of the survey data.

### *Chlamydia trachomatis* disease parameters

To measure the *Ct* distribution in the simulated population, we implemented the *Ct* infection process as described in Althaus et. al. [11] (Table 4), in which the rate of (unprotected) sexual contacts drops from once every 2 days to once every 7 days after the first two weeks of a partnership. The transmission rate of *Ct* in our simulations was set to 2.5% per partner per sexual contact, such that the average *Ct* prevalence in the age-group 18–44 matched the estimated *Ct* prevalence of 1.7% of the UK in the same age-group [11], [22]. A more in-depth study of the relationship between the decline of condom use and coital frequency during a partnership, and its (limited) effect on the distribution of *Ct* is presented as Supporting .

**Table 4:** *Chlamydia trachomatis* infection parameters.

Parameter	Category	Value
Incubation time		14 days
Fraction asymptomatic		75%
Duration	sympt. *Ct*	35 days
	asympt. *Ct*	433 days
Sexual activity during partnership	first 2 weeks	ave. of 1 time per 2 days
	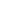 after 2 weeks	ave. of 1 time per 7 days
Transmission 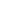 a	Kretzschmar model [7]	4.4% per sex act
	Current model	2.5% per sex act
Timestep of *Ct* transmission		1 day

a Transmission rate is fitted to match a *Ct* prevalence of 1.7% in the age-group 17–44, as per Althaus et. al. [11].

### Pair formation process

The model keeps track of the total number of partnerships between moderate individuals, between core-group members, and between core-group members and moderate individuals (Table 2). Every timestep of the model, any shortage of partnerships in the simulated population is supplemented by randomly sampling pairs of individuals from the population and attempting to form partnerships between them. Once enough new partnerships have been formed, the model moves one timestep ahead and repeats the same process. Because partnerships are continuously dissolved as time progresses, their total number constantly needs to be supplemented.

Not all individuals are available for sampling on a particular day. Individuals have to have the necessary free “capacity” for an additional partnership (taking into account that partnerships that are in their transitional concurrency phase no longer take up capacity). In addition, for moderate individuals there is an age- and gender-based probability that they are available for sampling that day (Table 2). This additional probability is not applied to core-group members. All individuals that are available for pair formation can be sampled to supplement the total number of partnerships in any of the appropriate combinations between core-group members and/or moderate individuals.

From the subset of the population that participates in the pair formation process on a given day, pairs of individuals are randomly sampled, and tested for three necessary conditions for pair formation:

1. The two individuals are of opposite gender
2. The two individuals are not already in a partnership with each other
3. Both individuals aim for a partnership of the same type (i.e. short-, medium-, or long-term partnerships, see methods)

If those conditions were all met, the probability of partnership formation was determined by the age disparity between the partners. To calculate this conditional probability, we use a folded cumulative normal distribution function (CDF) [9], [43] whose mean 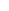 and variance 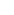 depend on the age 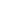 of the woman. The properties of this function are as follows: when the age disparity (male age - female age) corresponds to the mean of the folded CDF, the probability is 0.5, and any deviation from the mean results in a lower probability. To prevent unnecessary computations in the model by rejecting at least 50% of the partnerships, all probabilities generated by the folded CDF are multiplied by two. The equation that defines the mean age disparity, 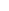, is described by
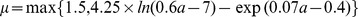
(1)


**Figure 9:**
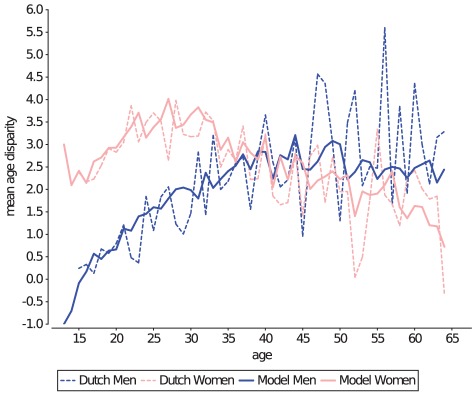
Age disparity of individuals in the network. The partnership formation probability function results in an age disparity in the model (solid lines) that closely matches the observed mean age disparity (dashed lines), as well as its standard deviation (not shown here) of the Dutch survey data.

This equation is plotted in Fig. 9A (solid pink line). Similarly, the variance 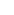 of the age disparity can be described by
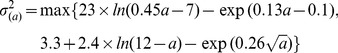
(2)


The shape and parameters of these equations were initially fitted to the *observed* age disparity and variance in the Dutch sexual survey data, but as these equations represent the *preferred* age disparity within partnerships of the simulated population, they needed to be subsequently adjusted (by trial and error) such that the resulting age disparity in the model matched that of the sexual survey data (Fig. 9). Among the adjustments were the addition of a necessary condition based on the age of male partner: for men below the age of 18, a partners' age should not be more than 2 years older than their own.

### Partnership type and duration

Based on the UK survey data on the duration of the second-most recent partnership (Fig. 5), partnerships in the model are categorized into three types (short, medium and long-term partnerships). Each type of partnership represents an exponential distribution with a minimum duration of 1 day, and a mean duration of 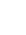 days (short), 250 days (medium), and 5880 days (long) (Table 2). The preferred duration of a partnership is determined by first picking the type of partnership from a ratio of 14 (short) to 13 (medium) to 12 (long), and subsequently sampling the exponential distribution associated with that type. As detailed in the previous section, a partnership will only be formed between two individuals if both select the same type of partnership.

### Capacity

The maximum number of partnerships that an individual can simultaneously maintain is described in the model by their sexual capacity, meaning that an individual with a sexual capacity of 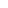 can maintain 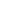 simultaneous partnerships. The sexual capacity of an individual reflects how much time, attention, money,etc he/she is willing to invest in partnerships [44].

In the model, the development of the sexual capacity of an individual during his/her life is stored in a vector that is constructed at its birth, by sampling from gamma distributions that determine the age at which an individual starts participating in the pair formation process (and thus when its capacity goes from 0 to 1), and if applicable, the onset, and duration of a period during which its capacity is larger than 1 (Table 1). Individuals with a capacity larger than 1 are part of the so-called “core-group” [23], [24] and are able to start and maintain multiple concurrent partnerships. The maximum capacity of a core-group member is a function of the onset of their core-group period, and the length of that period (Table 1). Core-group members that have a capacity of 5 or higher (about 15% of the core-group), will keep a higher base capacity of 2 after their core-group behaviour period.

Core-group members are not allowed to have more than two non-transitional (i.e. costly, see next section) long-term partnerships at the same time. This constraint makes it possible for some core-group individuals to accumulate up to 600 partnerships over their lifetime, as is observed in the empirical data (see results), and not become tied up in 5 long-term partnerships. When the capacity of an individual drops at the end of its core-group period, he/she will randomly break up a number of partnerships, until he/she is no longer over capacity.

### Transitional concurrency

Transitional concurrency (i.e. the period prior to the end of an existing partnership during which an individual acquires a new partnership [25]) is implemented as follows: partnerships that are near the predetermined end date of that partnership no longer carry a maintenance cost, and thus free up capacity for an individual. Transitional concurrency becomes a possibility during the last 15% of the duration of an existing partnership for men, and the last 10% for women. In effect, it allows individuals that have a maximum capacity of 1 to temporarily maintain two concurrent partnerships.

### Model initiation

The model has a burn-in period of 60 years, during which a stable sexual contact network is built up in the model population. Ten years prior to the end of the burn-in period, *Ct* is introduced in the simulated population by infecting 100 core-group individuals with asymptomatic *Ct*. After 35 years, the average *Ct* prevalence distribution is measured over a period of 15 years.

### Model implementation

The model is implemented in Clojure 1.21, a modern dialect of lisp (http://clojure.org). The source code of the model and an example of the sexual networks that are generated by the model are included as Protocol S1, and Dataset S1 & S2.

## Supporting Information

Dataset S1 Dataset S1 & S2 contain a textfile split in two parts, and together form an example of a simulated sexual network over a 100 year period. Each line in the dataset represents the life history of a single individual, and records the day they were born, how their sexual capacity developed (per year) during their lifetime, and with whom and from what day to what day they had a partnership. This sexual network was initialized with no existing partnerships, and thus needs about 60 years before it has stabilized. Linefeeds in the dataset are in linux format (LF and not CR-LF) and may need conversion on windows.
(BZ2)

Dataset S2 Dataset S1 & S2 contain a textfile split in two parts, and together form an example of a simulated sexual network over a 100 year period. Each line in the dataset represents the life history of a single individual, and records the day they were born, how their sexual capacity developed (per year) during their lifetime, and with whom and from what day to what day they had a partnership. This sexual network was initialized with no existing partnerships, and thus needs about 60 years before it has stabilized. Linefeeds in the dataset are in linux format (LF and not CR-LF) and may need conversion on windows.
(BZ2)

Figure S1The relationship between partnership duration, condom use and a proxy of coital frequency. The fraction of coital events during which condoms are used (black line) starts at 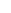
80%, and decreases to 16% as partnership duration increases (black circles [45]). The fraction of the population with an above-average (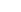) number of coital acts per month [46] (red squares) was used as a proxy for coital frequency (red line). The first datapoint in this series (the 100% at day 5) was not reported by Klusmann et. al [46], but is based on the assumption that partners would have had a coital event during the first five days of their partnership, and thus 100% would at 5 days have a coital frequency of 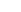 times per 4 weeks. The resulting relationship between partnership duration, the fraction of individuals not using condoms and a proxy of coital frequency is given by the orange line.(EPS)

Figure S2 The relationship between the number of *recent partners*, and the estimated number of sex acts in the last year, with (left) and without coital dilution (right). The figure shows the mean values, as well as the interquartile ranges of a single (typical) timepoint in the sexual contact network.
(EPS)

Figure S3 The relationship between the number of *recent partners*, and the number of days without partner. The figure shown here is a snapshot moment of the sexual contact network, and shows the mean values, as well as the interquartile ranges. Individuals with a high numbers of partners in the last year tend to be core-group members involved in concurrent partnerships, and have little or no days without sexual partners in a year, with the exception of individuals that during the year entered or left the core-group.
(EPS)

Figure S4 The effect of different assumptions on the *Ct* prevalence distribution. Compared to the main model (orange blocks, zoomed in version of Fig. 8., main manuscript), the various scenarios tested in this supporting text had a limited effect on the *Ct* prevalences of those with 3, 4 or 5+ sexual partners in the last year, and did not result in a pattern where for an overall *Ct* prevalence of 1.7%, the highest prevalence would be found in the group with 3 recent partners (as observed in the UK). The scenario with coital dilution includes a constant *Ct* transmission rate per day, and the immunity scenarios include both the constant transmission rate and coital dilution effects (of strength 0.7).
(EPS)

Protocol S1 Contains the source code of the individual-based model, together with the directory structure, documentation and the tools necessary to download the libraries and to create standalone JAR files with which to generate sexual network datafiles.
(BZ2)

Text S1 Contains a more in-depth study of the relationship between the decline of condom use and coital frequency during a partnership, and its (limited) effect on the distribution of *Ct*, as well as our exploratory study of two possible mechanisms that might explain the observed *Ct* prevalence distribution, in which *Ct* prevalence after an initial increase, appears to decrease with an increase in sexual activity [11].
(PDF)
