# Supplementary material for: Determinants of Sexual Network Structure and Their Impact on Cumulative Network Measures
Source: PLoS Comput Biol. 2012 Apr 26;8(4):e1002470. doi: 10.1371/journal.pcbi.1002470 (PMC3343090; doi:10.1371/journal.pcbi.1002470)
Supplement: Text S1 — Contains a more in-depth study of the relationship between the decline of condom use and coital frequency during a partnership, and its (limited) effect on the distribution of Ct, as well as our exploratory study of two possible mechanisms that might explain the observed Ct prevalence distribution, in which Ct prevalence after an initial increase, appears to decrease with an increase in sexual activity [11]. (PDF) [file pcbi.1002470.s008.pdf]

Supporting Text S1

Condom use and coital frequency decrease at a similar rate during partnerships

Both condom use and coital frequency are expected to decrease during a partnership, but it is not well established whether they decrease at a similar rate, such that the opportunity for transmitting a particular sexually transmittable disease (STD) can be said to remain constant *per day* throughout the partnership. An indication that such an assumption is justified can be found by combining data on the decline of condom use as a function of partnership duration [1], and the decline in coital frequency as a function of partnership duration [2]. After performing some arithmetical conversions on these two datasets (see later in this section), we could fit two exponential curves of the form $\mathrm{y} = \mathrm{a} + \mathrm{b}\exp^{cx}$ through the datasets (Fig. S1, black and red lines). The resulting relationship between not using condoms, and coital frequency (Fig. S1, orange line) supports the idea that the frequency of unprotected sex acts during a partnership can be approximated by a constant transmission rate per day.

The average condom use percentage at different stages in a partnership was not directly available in the communication of Kuyper et. al. [1], but was derived from the following observations that were reported in the communication:

- 56% of the surveyed population in short-term partnerships indicated that they always used a condom during vaginal intercourse, and 14% that they never used a condom.
- Consistent condom use is at its highest early during partnerships (56%), but decreases rapidly: 8% of the couples stops using condoms consistently during the first month, a further 35% stops using condoms consistently between 1-3 months, and another 35% between 3-6 months. The remaining 22% took longer than 6 months before reducing their condom use.

Assuming that over time individuals shift from consistent condom, to inconsistent condom use, to no condom use, and that inconsistent condom use can be summarized as using condoms during half the sexual contacts, and that the decline in inconsistent condom use follows the same decline as that in consistent condom use, the average decline in condom use would be as follows:

- on average 71% of the sexual contacts during short-term partnerships (which in the sexual network simulation model are on average 13.33 days old) are with condom use.

- at 1 month, consistent condom use and irregular condom use will have decreased by 4.5%, compared to the rate of condom use at 13.33 days, meaning that on average 69% of the sexual contacts in partnerships of 1 month old are estimated to make use of condoms, i.e. $(0.56 * (1 - 0.045)) * 1 + ((0.56 * 0.045) + (0.3 * (1 - 0.045))) * 0.5 + ((0.3 * 0.045) + 0.14) * 0 = 69\%$.
- at 3 months, 35% of the original 56% are assumed to be inconsistently using condoms, 4.5% of the original 56% of consistent condom-users are assumed to have passed through a phase of inconsistent condom use and have now stopped using condoms at all, and 39.5% of the 30% that initially were inconsistently using condoms have also stopped using condoms at all. Therefore the average condom use at 3 months is in 53% of the sexual contacts, i.e. $(0.56 * (1 - 0.045 - 0.35)) * 1 + ((0.56 * 0.35) + 0.3 * (1 - 0.045 - 0.35))) * 0.5 + (0.3 * (0.045 + 0.35) + 0.14) * 0) = 53\%$.
- continuing in this manner, the average condom use at 6 months is 28%.
- As 29% of the 56% report that are consistently using condoms are doing so to prevent pregnancy, we assume that consistent condom use will not fall below 16%, but that inconsistent condom use will fall to 0%, leading to the final datapoint of 16% condom use.

These numbers form the datapoints in Fig. S1 that represent the decline in condom use with partnership duration.

Decreased *Ct* prevalence at higher levels of sexual activity

The remarkable drop in *Ct* prevalence for higher levels of recent sexual activity (see Althaus et. al. [3], and Fig. 8, main article) has been explained in several ways. We will investigate two options here in more detail, namely 1) that the drop in *Ct* prevalence at higher levels of recent sexual activity is caused by a reduced coital frequency per partner in individuals with concurrent partnerships [4, 5], and 2) that it reflects the effects of acquired immunity to re-infection after naturally resolving an asymptomatic *Ct* infection [6–8].

Prior to implementing some form of coital dilution in the model, we made effort to compare the relationship within the simulation model between the yearly coital frequency (both protected and unprotected by condom use) and the number of partners in the last year. This allowed us to investigate whether the model output corresponded to an observed negative correlation between yearly coital frequency and the recent number of partners [4]. The equation that we used to describe the coital acts

per day and per partnership for a person, given the duration d of that partnership can be formulated as $\frac{0.38+0.62\exp^{-0.0038d}}{1.6}$, and is the fit of the proxy of coital frequency used in Fig. S1, scaled by a factor 1.6, such that the coital acts per year in the model for persons age 16-31 with 1 partnership matches that of the study population of Nordvik et. al. [4].

Interestingly, even though in our model the number of sex acts per day increases linearly with the number of current sexual partners, the relation between coital frequency and the **recent** number of partners is similar to that of the Swedish population [4]: the model results show a slow increase in coital frequency per year for men (compared to a constant coital frequency in Sweden), as their number of recent partners increases to up to 4 partners (Fig. S2), and an almost constant coital frequency for women up to 6 partners (compared to a declining coital frequency in Sweden).

We implemented a simple form of coital dilution in which the daily coital frequency (and thus the daily transmission rate) per partner c is reduced in response to the number of **current** partners n of the an individual, such that $c_n = \frac{c_1}{n^{0.7}}$. c_1 is the daily *Ct* transmission rate for individuals with 1 partner, where 0.7 reflects the strength of the coital dilution. We then re-adjusted the *Ct* transmission chance per day, such that the overall *Ct* prevalence in the age-range 16-45 again matched that of the UK (as we did in the main manuscript). Note that a more correct form of coital dilution would be to take into account the coital dilution of both partners within a partnership and make an informed decision on the coital frequency per partnership. However in this exploratory study we keep to the simple form described here, in which effectively only the number of partners of the infected individual influence the coital dilution within a partnership.

The above form of coital dilution results in a good fit between the model results and the observed coital frequency for individuals with 1 partnership in the last year, as well as those with 6+ partnerships in the last year. However, coital dilution did not result in the observed negative correlation between coital frequency and number of partners for 2-5 partners in the last year [4]. Rather, the effect of coital dilution appeared to be mainly limited to those individuals with 6+ partners in the last year, i.e. the core-group individuals. We can understand the lack of effect of coital dilution on individuals with < 6 partners per year by realizing that individuals with a modest number of partnerships are likely to be regular, non-coregroup individuals that are involved in a series of short- and medium-term partnerships: these individuals are only affected by the effects of coital dilution during the short periods of transitional concurrency. Support for this explanation is evident from the relation between the number of days

that individuals are single and their number of partners in the last year (Fig. S3): individuals with a limited number of partners (2-4 for men, 2-8 for women) are found to be single for at least part of the year (Fig. S3), which reduces their yearly number of coital acts compared to individuals with 1 long-term partnership. Given these results, we speculate that the difference between the sexual contact network simulated in this paper, and that of the Swedish population might be a lower level of transitional concurrency and/or a larger positive gap length between partnerships in the latter. Such a difference would result in a negative relation between the recent number of partnerships and the number of coital acts per year for individuals engaged in serial monogamy.

The effect on the distribution of *Ct* of implementing a constant per-day transmission rate, rather than a decline in transmission rate after the first two weeks of a partnership (as was the case in Althaus et. al. [3] and the main model), appears to be negligible (Fig. S4, red line versus the orange blocks). Implementing coital dilution does have an effect on the *Ct* distribution: compared to the distribution in the main model, it lowers the prevalence of *Ct* in those with 5+ partners in the last year and increases the Ct prevalence in those with 2, 3 or 4 partners in the last year (Fig. S4, blue line). However, it does not lead to the pattern observed in the Natsal UK sexual survey, in which *Ct* prevalence peeks in individuals with 3 partners per year. A further increase in the intensity of coital dilution (from 0.7 to 1.3) did not qualitatively change these results.

Acquired immunity after asymptomatic *Ct* infections

A second mechanism that is suggested as a possible cause of the lower than expected prevalence in those individuals with more than 3 partners in the last year would be some mechanism of acquired immunity after prolonged or frequent exposure to *Ct*. We implement two scenarios of acquired immunity - one where an asymptomatic episode of *Ct* confers a lifelong immunity against re-infection, and one where the same episode confers immunity for 5 years. Symptomatic episodes are of short duration in the model, and expected to trigger health-care search and treatment in the infected individual, and thus not lead to acquired immunity. Both scenarios are combined with a constant daily transmission chance of *Ct* within partnerships, and a coital dilution of 0.7, fitted to the Swedish population [4].

We find that in the scenario of lifelong immunity there is no realistic transmission rate of *Ct* possible that would result in an average *Ct* prevalence of 1.7%. At a transmission rate of 100% per day (for individuals not affected by coital dilution), a population prevalence of at most 1.4% is reached (Fig. S4,

green line). The *Ct* prevalence of individuals with 5+ partnerships per year is substantially reduced (11%) compared to the prevalence of the same group in the main model (22%), but is still higher than in individuals with 3 or 4 partners in the last year. In the scenario where immunity lasts only 5 years after a prolonged asymptomatic *Ct* infection, a transmission chance of 11% per day suffices to maintain a population prevalence of 1.7%. However, this scenario does not lead to the substantial reduction of *Ct* prevalence observed in individuals with 5+ partnerships per year, as was the case in the scenario with lifelong immunity.

In conclusion, neither our simple implementations of coital dilution, nor some sort of acquired immunity appear able to cause a decline in *Ct* prevalence in individuals with 4+ partnerships in the last year, compared to those with 3+ partnerships.

References

1. Kuyper L, Bakker F, Zimbile F (2008) Veilig vrijen bij jongeren. De stand van zaken 2007 en de ontwikkeling sinds 1997. Rutgers Nisso Groep. URL `www.rutgerswpf.nl/sites/default/files/Jongerenmonitor%202009.pdf`.

2. Klusmann D (2002) Sexual motivation and the duration of partnership. Arch Sex Behav 31: 275–87.

3. Althaus CL, Turner KME, Schmid BV, Heijne JCM, Kretzschmar M, et al. (2011) Transmission of Chlamydia trachomatis through sexual partnerships: a comparison between three individual-based models and empirical data. J R Soc Interface .

4. Nordvik MK, Liljeros F (2006) Number of sexual encounters involving intercourse and the transmission of sexually transmitted infections. Sex Transm Dis 33: 342–9.

5. Sawers L, Isaac AG, Stillwaggon E (2011) HIV and concurrent sexual partnerships: modelling the role of coital dilution. J Int AIDS Soc 14: 44.

6. Brunham R, Kimani J, Bwayo J, Maitha G, Maclean I, et al. (1996) The epidemiology of Chlamydia trachomatis within a sexually transmitted diseases core group. J Infect Dis 173: 950.

7. Brunham RC, Pourbohloul B, Mak S, White R, Rekart ML (2005) The unexpected impact of a Chlamydia trachomatis infection control program on susceptibility to reinfection. J Infect Dis 192: 1836–44.

8. Batteiger B, Xu F, Johnson R, Rekart M (2010) Protective Immunity to Chlamydia trachomatis Genital Infection: Evidence from Human Studies. J Infect Dis 201: 178–189.
